# Maternal Baseline Characteristics and Perinatal Outcomes: The Tohoku Medical Megabank Project Birth and Three-Generation Cohort Study

**DOI:** 10.2188/jea.JE20200338

**Published:** 2022-02-05

**Authors:** Junichi Sugawara, Mami Ishikuro, Taku Obara, Tomomi Onuma, Keiko Murakami, Masahiro Kikuya, Fumihiko Ueno, Aoi Noda, Satoshi Mizuno, Tomoko Kobayashi, Yohei Hamanaka, Kichiya Suzuki, Eiichi Kodama, Naho Tsuchiya, Akira Uruno, Yoichi Suzuki, Osamu Tanabe, Hideyasu Kiyomoto, Akito Tsuboi, Atsushi Shimizu, Seizo Koshiba, Naoko Minegishi, Soichi Ogishima, Gen Tamiya, Hirohito Metoki, Atsushi Hozawa, Nobuo Fuse, Kengo Kinoshita, Shigeo Kure, Nobuo Yaegashi, Shinichi Kuriyama, Masayuki Yamamoto

**Affiliations:** 1 Tohoku Medical Megabank Organization, Tohoku University, Sendai, Japan; 2 Graduate School of Medicine, Tohoku University, Sendai, Japan; 3 Tohoku University Hospital, Sendai, Japan; 4 School of Medicine, Teikyo University, Tokyo, Japan; 5 International Research Institute of Disaster Science, Tohoku University, Sendai, Japan; 6 Ageo Central General Hospital, Saitama, Japan; 7 Radiation Effects Research Foundation, Hiroshima, Japan; 8 Graduate School of Dentistry, Tohoku University, Sendai, Japan; 9 Iwate Tohoku Medical Megabank Organization, Disaster Reconstruction Center, Iwate Medical University, Iwate, Japan; 10 The Advanced Research Center for Innovations in Next-Generation Medicine, Tohoku University, Sendai, Japan; 11 Center for Advanced Intelligence Project, RIKEN, Tokyo, Japan; 12 Faculty of Medicine, Tohoku Medical Pharmaceutical University, Sendai, Japan; 13 Graduate School of Information Sciences, Tohoku University, Sendai, Japan

**Keywords:** baseline profile, perinatal outcome, birth cohort, developmental origins of health and disease

## Abstract

**Background:**

The Tohoku Medical Megabank Project Birth and Three-Generation Cohort Study was launched in 2013 to evaluate the complex interactions of genetic and environmental factors in multifactorial diseases. The present study describes the maternal baseline profile and perinatal data of participating mothers and infants.

**Methods:**

Expectant mothers living in Miyagi Prefecture were recruited from obstetric facilities or affiliated centers between 2013 and 2017. Three sets of self-administered questionnaires were collected, and the medical records were reviewed to obtain precise information about each antenatal visit and each delivery. Biospecimens, including blood, urine, umbilical cord blood, and breast milk, were collected for the study biobank. The baseline maternal sociodemographic characteristics, results of screening tests, and obstetric outcomes were analyzed according to the maternal age group.

**Results:**

A total of 23,406 pregnancies involving 23,730 fetuses resulted in 23,143 live births. Younger maternal participants had a tendency toward a higher incidence of threatened abortion and threatened premature labor, while older age groups exhibited a significantly higher rate of low lying placenta, placenta previa, gestational diabetes, and hypertensive disorders of pregnancy.

**Conclusions:**

The present study clearly shows the distribution of maternal baseline characteristics and the range of perinatal outcomes according to maternal age group. This cohort study can provide strategic information for creating breakthroughs in the pathophysiology of perinatal, developmental, and noncommunicable diseases by collaborative data visiting or sharing.

## INTRODUCTION

Noncommunicable diseases (NCDs) are defined as chronic diseases caused by the interaction of genetic, physiologic, environmental, and behavioral factors.^1^^,^^2^ Accumulating evidence for the developmental origins of health and disease (DOHaD)^3^^,^^4^ suggests that the intrauterine environment plays an important role of the onset of NCDs, mainly through effects on the maternal or placental factors. Affected fetuses develop NCDs later in life, including developmental, psychologic, cardiovascular, endocrinologic, and respiratory disorders.^1^^,^^2^ To uncover the etiology and pathophysiology of these NCDs, several large-scale birth-cohort studies have been established.^5^^–^^10^ Recent cohort studies have been focused on improving statistical power by maximizing the number of participants or by adding familial information from several generations.^11^^,^^12^

In 2011, the Great East Japan Earthquake (GEJE) and Tsunami destroyed wide areas of the Tohoku region, including Miyagi, Iwate, and Fukushima prefectures. Nine years have passed since the earthquake, and the official toll is 19,689 people confirmed dead and 2,563 still missing.^13^ Perinatal medical support systems were also affected by the GEJE,^14^ leading to concerns about short- and long-term health problems in mothers and infants.^15^^,^^16^ The Tohoku Medical Megabank Project (TMM), established by the Tohoku Medical Megabank Organization (ToMMo) and the Iwate Tohoku Medical Megabank Organization, was started in February 2012 to provide maximal efforts to reconstruct damaged health care services in areas severely affected by the GEJE.^17^^,^^18^ The TMM Birth and Three-Generation Cohort Study (BirThree Cohort Study) was launched in 2013 to evaluate the complex interactions of genetic and environmental factors using information about in utero and subsequent pediatric exposures, and about maternal, pediatric, and family outcomes by evaluating a birth cohort and members of three generations.^19^ The detailed study design has been reported elsewhere^19^; briefly, expectant mothers were recruited from obstetric facilities, with subsequent recruitment of their family members.

Advanced maternal age at delivery has been raised as one of serious issues in perinatal medicine. In the last decades, rate of pregnancies with advanced age has increased steadily in developed countries.^20^ Recent reports suggested that advanced maternal age was related to obstetric outcomes such as preeclampsia, gestational diabetes, placenta previa and fetal growth restriction.^21^ However, landscape obstetric outcomes in large scale cohort studies have not been precisely demonstrated, especially in Japan.

We aim to analyze the maternal baseline information and perinatal outcomes in mothers and newborns by maternal age group. The present paper would provide strategic information for future integrative association studies on a variety of perinatal, developmental, health, and disease issues, including NCDs.

## METHODS

### Study participants

The detailed protocol of the TMM BirThree Cohort Study has been reported elsewhere.^19^ A sample size of approximately 70,000 participants was calculated based on the requirements for a multipurpose research platform that included genetic analysis,^17^ the number of births in Miyagi Prefecture, the estimated number of incident disease cases, feasibility and costs.^19^ In the TMM BirThree Cohort Study, all obstetric facilities in the prefecture were asked to participate for the study. And seven community support centers, for the voluntary admission-type recruitment and health assessment of participants, were newly established to cover wide areas of inland and coast side of Miyagi Prefecture.^17^ And pregnant women were recruited in an early stage of pregnancy from approximately 50 obstetric clinics and hospitals, and from 7 community support centers in Miyagi Prefecture, between 2013 to 2017. Several women were recruited during multiple pregnancies across the study period, as previously reported.^19^ Certified genome medical research coordinators (GMRCs) provided precise information on the TMM BirThree Cohort Study to potential participants and obtained written informed consent from each participant.^22^

### Assessments during the prenatal period

Self-administered questionnaires were designed by referring to ongoing cohort studies.^19^ Participants were provided with three questionnaires: at enrolment, during midpregnancy, and 1 month after delivery.^19^ The first questionnaire included baseline and family information, reproductive and medical history, medication history, and assessments of smoking and alcohol consumption, physical activity, sleep, employment history, mental health, and eating habits and nutrition. The second questionnaire included assessments of the participants’ living environment, social connections, socioeconomic status, and personality. The third questionnaire included assessments of mental health and information about the newborn, including its living environment.^19^ Questionnaires were also administered to family participants, including fathers, grandparents, great-grandparents, and siblings of the included infants.

### Medical records

Medical and health care information was collected by transcribing medical records from obstetric clinics and hospitals. In Japan, health checkups for pregnant women are supported by the government, and 14 perinatal visits are designated to check maternal body weight, blood pressure, and urinalysis, and to conduct necessary screening for blood type, irregular antibodies, infection, cervical cancer, and anemia, and to assess the fetal heart rate.^23^ Fetal ultrasound examination included measurement of the biparietal diameter, abdominal circumference (or fetal trunk area), femur length, estimated fetal body weight, and amniotic fluid index (or vertical pocket depth). The records from each antenatal visit and the birth information were collected by GMRCs and transferred to the TMM integrated database with strict security precautions.^24^

Maternal baseline profiles were taken from the medical records or questionnaire data and included maternal age, parity, prepregnancy body mass index (BMI), smoking status, alcohol consumption, any fertility treatment for the current pregnancy, educational background, occupation, and annual household income. Information on obstetric complications were collected from the medical records and included hyperemesis, threatened abortion, threatened premature labor, placenta previa, hypertensive disorders of pregnancy (HDP), gestational diabetes mellitus (GDM), placental abruption, nonreassuring fetal status (NRFS), fetal growth restriction (FGR), intrauterine fetal demise (IUFD), premature rupture of membranes (PROM), chorioamnionitis, oligohydramnios, polyhydramnios, atonic bleeding, and the hemolysis, elevated liver enzymes, and low platelet count (HELLP) syndrome. The chorionicity of multiple gestations was also recorded. Clinical diagnosis was transcribed from medical record data and doctor’s diagnosis was conducted according to the Japan Society of Obstetrics and Gynecology Guideline.^25^

Birth information, transcribed from medical record data, included the mode of delivery, blood loss within 2 hours after delivery, status of labor induction, and history of blood transfusion. The birth profile of newborns included the gestational age at delivery, sex, birth weight, birth height, head circumference, chest circumference, placental weight, Apgar scores, pH of the umbilical artery (UApH), and admission to the neonatal intensive care unit (NICU). Medical record data and questionnaire data both collected for each participant and stored separately. When the medical record data and questionnaire data did not match, data cleaning committee in our organization would add flags on each data and decided appropriate data for the analysis.

### Biospecimens

Biospecimens were collected for the TMM biobank.^26^ Blood and urine samples were collected at same time as the three questionnaires were administered. Additionally, umbilical cord blood was collected at birth, and breast milk was collected at the routine visit 1 month after delivery. Family members were also asked to provide blood and urine samples. Laboratory data were obtained from the medical records and including screening syphilis testing (rapid plasma reagin [RPR], treponema pallidum hemagglutination [TPHA]), and testing for hepatitis B virus (HBV), hepatitis C virus (HCV), HIV, rubella, human t-cell leukemia virus type 1, toxoplasmosis, and herpes simplex virus (HSV). As previously reported, bioresources were anonymized and transferred to the TMM biobank under an extremely high level of security.^24^^,^^26^

### Ethical issues

As previously reported, the protocol for the TMM BirThree Cohort Study was approved by the Ethics Committee of ToMMo (2013-1-103-1).^19^ The present study was conducted in accordance with the Declaration of Helsinki^27^ and the Ethical Guidelines for Human Genome/Gene Analysis Research.^28^ Broad and continuing consent was required for participation in the cohort.^17^ Written informed consent was obtained from all participants as described above. For participants lacking the ability to understand the study protocol, informed consent from the participant’s guardian was obtained.

### Statistical analysis

We analyzed the characteristics of mothers and newborns by maternal age group: ≤24 years of age, 25–29 years of age, 30–34 years of age, 35–39 years of age, and ≥40 years of age. Maternal characteristics, results of maternal infection screening tests, and maternal and birth outcomes stratified by maternal age were compared using Cochran-Armitage trend test and linear regression analysis, as appropriate.

All data fixed in our database as of February 1, 2019, were used in the present study.

## RESULTS

The final number of participants in the BirThree Cohort Study was 22,493 mothers, 23,143 newborns, 9,459 fathers, 5,941 grandparents of mothers, 2,117 grandparents of fathers, and 1,553 other family members. Selected perinatal baseline data were reported separately.^19^ Maternal baseline characteristics by age group are shown in Table 1. Of the 23,406 pregnancies, 9.4% were in women ≤24 years of age, 27.5% were in women 25–29 years of age, 35.8% were in women 30–34 years of age, 21.5% were in women 35–39 years of age, and 5.9% were in women ≥40 years of age. There was a high prevalence of prepregnancy BMI <18.5 kg/m^2^ group in younger participants (*P* < 0.0001). Some women required fertility treatment to achieve the current pregnancy; ovulation induction was used in 1.2%, artificial insemination in 0.9%, in vitro fertilization (IVF) in 2.9%, and intracytoplasmic sperm injection (ICSI) in 1.2%. The older age groups tended to use more AIH, IVF, and ICSI (*P* < 0.0001). Higher education levels (≥13 years) were noted in 66.9% of participants, and 73.3% had an occupation. The annual household income was 2 to 4 million Japanese yen per year in 32.6%, and 4 to 6 million in 32.5%.

**Table 1.** Maternal baseline characteristics

Variables	*n* ^a^	Total	Age at enrollment, years					*P* for trend

			≤24	25–29	30–34	35–39	≥40	

		%	%	%	%	%	%	
Number	23,406		2,192	6,427	8,370	5,027	1,390	

Parity	23,068							
0		46.9	70.9	55.3	41.4	35.9	42.3	<0.0001
1		35.4	25.0	32.5	38.3	39.0	33.7	<0.0001
≥2		17.7	4.1	12.2	20.3	25.2	23.9	<0.0001

Prepregnancy BMI, kg/m^2^	23,406							
<18.5		20.1	28.4	21.1	17.9	16.0	29.9	<0.0001
18.5 to <25		68.8	63.3	68.4	71.0	70.7	58.9	0.25
25 to <30		8.3	6.3	7.8	8.0	10.4	8.1	<0.0001
≥30		2.8	2.0	2.6	3.1	2.9	3.1	0.022

Smoking status	22,129							
Never smoker		59.8	54.9	62.0	61.5	56.1	60.0	0.12
Past smoker before pregnancy		23.5	15.0	19.2	24.2	30.4	26.9	<0.0001
Past smoker after pregnancy		14.2	26.0	16.3	12.0	11.3	10.3	<0.0001
Current smoker		2.5	4.1	2.4	2.4	2.2	2.8	0.0056

Alcohol consumption	22,151							
Current drinker		19.4	14.0	18.7	20.1	21.4	19.6	<0.0001
Past drinker		34.6	34.5	34.7	34.0	34.4	39.9	0.16
Never drinker		40.3	45.5	41.3	40.0	38.5	34.4	<0.0001
Constititionally never drinker		5.8	5.9	5.4	5.9	5.8	6.1	0.53

Fertility treatment^b^	23,083							
Natural pregnancy		93.4	99.5	97.5	94.0	87.8	78.0	<0.0001
Ovulation Induction		1.2	0.2	1.2	1.4	1.2	0.8	0.060
AIH		0.9	0.0	0.3	1.0	2.0	1.3	<0.0001
IVF		2.9	0.0	0.5	2.3	6.1	12.9	<0.0001
ICSI		1.2	0.0	0.3	0.9	2.4	5.7	<0.0001

Educational background	13,721							
Elementary/junior high school		2.6	9.6	2.7	2.3	1.2	1.7	<0.0001
High school		30.2	57.1	33.1	26.4	25.4	26.5	<0.0001
Vocational college		26.7	19.3	28.1	28.1	25.6	25.4	0.42
Junior College and Technical College		11.9	5.3	8.8	10.1	17.8	18.5	<0.0001
University		26.2	8.2	26.1	29.7	26.6	25.1	<0.0001
Graduate School		2.1	0.1	1.1	2.5	3.1	2.7	<0.0001
Other		0.2	0.4	0.2	0.2	0.3	0.1	0.76

Occupation	18,885							
Housewife or unemployed		26.2	23.2	23.8	26.7	28.6	32.0	<0.0001
Employed		73.3	73.4	76.1	73.2	71.2	68.0	<0.0001
Student		0.4	3.4	0.1	0.1	0.2	0.0	<0.0001

Household income, million Japanese yen/year	20,579							
<2		4.2	11.8	4.5	3.0	3.0	3.3	<0.0001
2 to <4		32.6	51.3	39.1	29.7	24.7	21.0	<0.0001
4 to <6		32.5	24.4	32.4	34.9	32.7	27.6	0.0036
6 to <8		18.4	7.2	15.3	19.8	22.6	26.6	<0.0001
8 to <10		7.4	2.5	5.5	8.0	9.6	11.6	<0.0001
≥10		4.9	2.9	3.2	4.6	7.4	9.9	<0.0001

AIH, artificial insemination with husband’s semen; BMI, body mass index; ICSI, intracytoplasmic sperm injection; IVF, in vitro fertilization (conventional).

^a^Missing values were excluded.

^b^Multiple choice allowed and excluded in this table.

Table 2 shows the results of pregnancy-related infectious disease screening. The rates of positive results were: RPR, 0.27%; TPHA, 0.23%; HBV, 0.26%; HCV, 0.22%; HIV, 0.1%; rubella X256, 5.41%, rubella, X512 1.5%, rubella X1024, 0.1%; and HITLV-1, 0.34%. Some study participants opted to be tested for HSV and toxoplasmosis, with positive rates of 3.4% and 8.1%, respectively. Older participants exhibited a higher rate of HBs Ag positive and Rubella Ab ≥X 64 (*P* < 0.0001).

**Table 2.** Maternal infectious disease screening

Variables	*n*	Total		Age at enrollment, years										*P* for trend

				≤24		25–29		30–34		35–39		≥40		

		*n*	(%)	*n*	(%)	*n*	(%)	*n*	(%)	*n*	(%)	*n*	(%)	
RPR	19,062													
+		51	0.27	1	0.06	12	0.23	19	0.28	16	0.38	3	0.31	0.040
−		19,011	99.7	1,808	99.9	5,172	99.8	6,863	99.7	4,199	99.6	969	99.7	

TPHA	19,071													
+		44	0.23	6	0.32	10	0.19	17	0.25	7	0.17	4	0.44	0.65
−		19,027	99.7	1,846	99.7	5,247	99.81	6,869	99.75	4,153	99.83	912	99.56	

HBs Ag	21,188													
+		56	0.26	0	0	8	0.14	19	0.25	20	0.44	9	0.88	<0.0001
−		21,132	99.7	2,051	100.0	5,869	99.9	7,638	99.8	4,565	99.6	1,009	99.1	

HCV Ab	20,813													
+		46	0.22	2	0.10	15	0.26	13	0.17	14	0.31	2	0.20	0.36
−		20,767	99.8	2,025	99.9	5,759	99.7	7,478	99.8	4,507	99.7	998	99.8	

HIV Ab	21,005													
+		21	0.10	3	0.15	7	0.12	5	0.07	4	0.09	2	0.20	0.72
−		20,984	99.9	2,034	99.9	5,829	99.9	7,558	99.9	4,569	99.9	994	99.8	

Rubella Ab	15,646													
	<X 8	251	1.60	49	3.09	107	2.40	70	1.24	20	0.61	5	0.72	<0.0001
	X 8	1,980	12.65	406	25.57	808	18.14	446	7.92	258	7.86	62	8.99	<0.0001
	X 16	2,635	16.84	374	23.55	932	20.92	764	13.57	467	14.22	98	14.20	<0.0001
	X 32	3,870	24.73	387	24.37	1,175	26.37	1,362	24.19	797	24.28	149	21.29	0.030
	X 64	3,634	23.23	259	16.31	853	19.15	1,525	27.09	821	25.01	176	25.51	<0.0001
	X 128	2,178	13.92	88	5.54	408	9.16	950	16.87	602	18.34	130	18.84	<0.0001
	X 256	846	5.41	17	1.07	134	3.01	401	7.12	244	7.43	50	7.25	<0.0001
	X 512	234	1.50	8	0.50	34	0.76	104	1.85	69	2.10	19	2.75	<0.0001
	X 1024	18	0.1	0	0.0	4	0.1	8	0.1	5	0.2	1	0.1	0.14

HTLVAb	19,423													
+		67	0.34	4	0.21	16	0.30	32	0.46	11	0.26	4	0.43	0.51
−		19,356	99.7	1,867	99.8	5,387	99.7	6,964	99.5	4,212	99.7	926	99.6	

HSV	529													
+		18	3.40	0	0	4	2.55	6	3.21	4	4.44	4	16.00	0.0023
−		511	96.6	70	100.0	153	97.5	181	96.8	86	95.6	21	84.0	

Toxoplasmosis	5,020													
+		407	8.11	33	6.69	112	7.63	159	8.81	80	7.77	23	10.13	0.18
−		4,613	91.9	460	93.3	1,355	92.4	1,645	91.2	949	92.2	204	89.9	

HBsAg, hepatitis B surface antigen; HCV Ab, hepatitis C antibody; HIV Ab, HIV antibody; HSV, herpes simplex virus; HTLV-1Ab, human t-cell leukemia virus type 1; RPR, rapid plasma regain test; Rubella Ab, Rubella antibody; TPHA, Treponema pallidum hemagglutination test.

**Figure 1.**
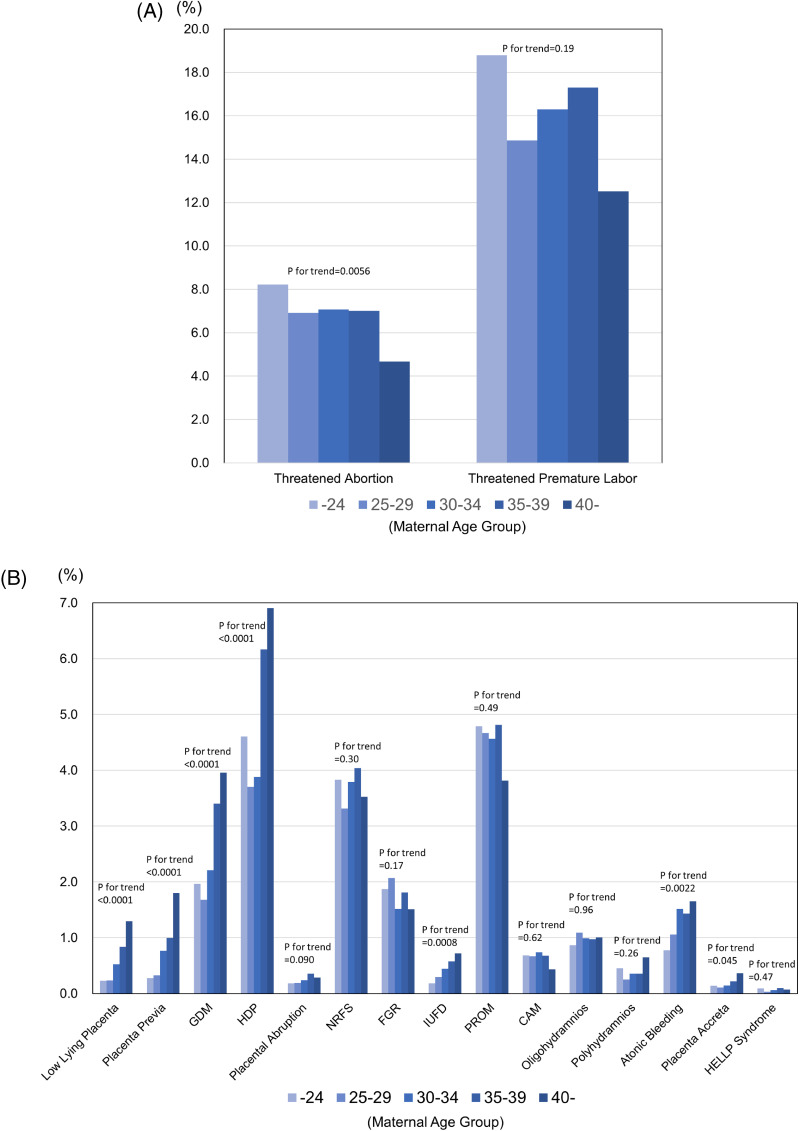
Prevalence of pregnancy-related complications **A**) by age group (*n* = 23,406) and **B**) by age group (*n* = 23,406). CAM, chorioamnionitis; FGR, fetal growth restriction; GDM, gestational diabetes mellitus; HDP, hypertensive disorders of pregnancy; IUFD, intrauterine fetal demise; NRFS, nonreassuring fetal status; PROM, premature rupture of membranes; HELLP, hemolysis, elevated liver enzymes, and low platelet count.

Figure 1 shows the prevalence of major pregnancy-related complications. Common obstetric complications were threatened premature labor (16.1%), threatened abortion (7.0%), HDP (4.6%), NRFS (3.7%), and FGR (1.8%). Younger participants tended to have a higher incidence of threatened abortion and threatened premature labor (Figure 1A), while older age groups had a significantly higher rate of low lying placenta, PP, GDM, and HDP (*P* < 0.0001) (Figure 1B).

**Figure 2.**
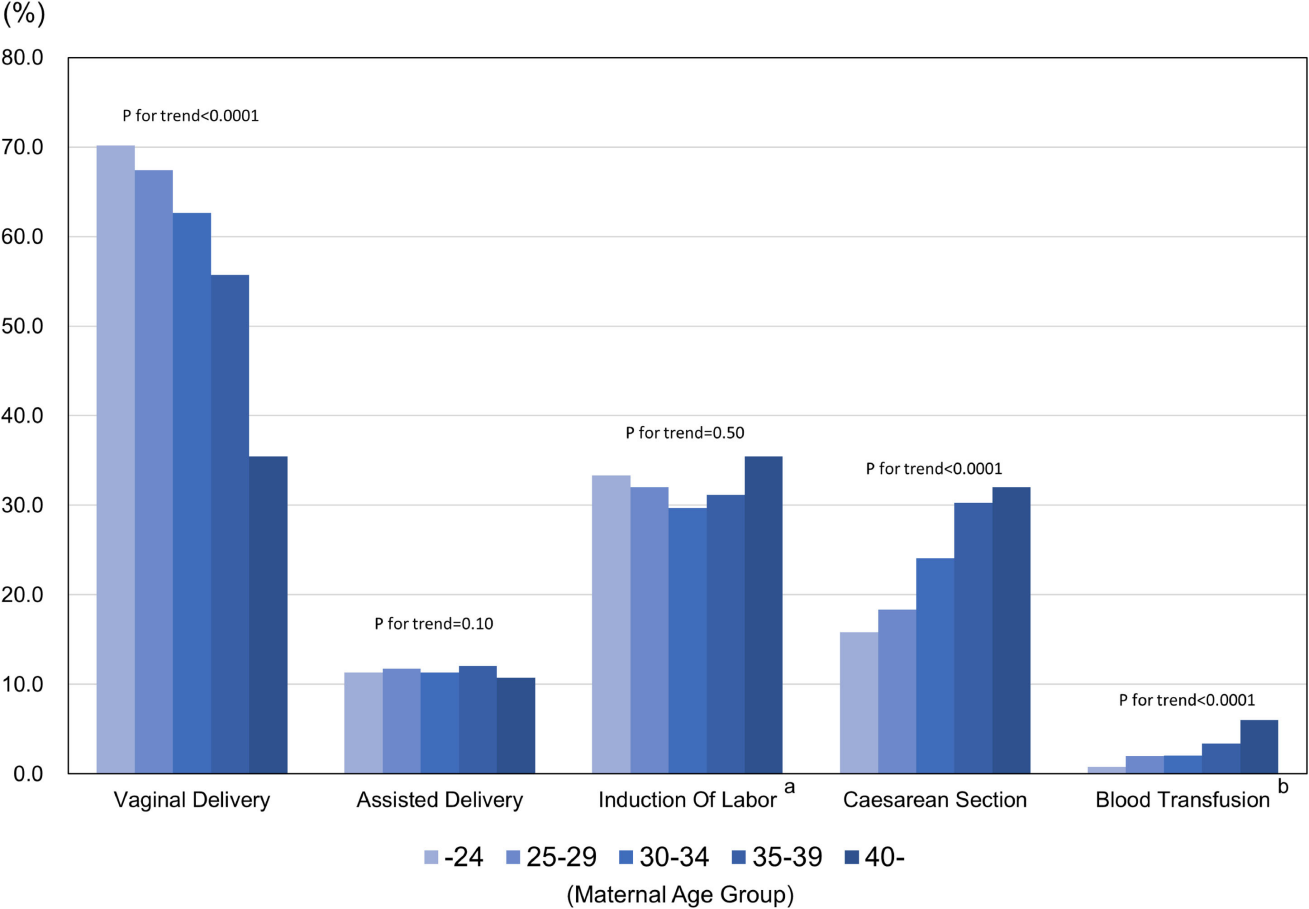
Delivery information according to age group (*n* = 23,406). ^a^Information on induction of labor was collected from 18,456 medical records; ^b^Information on blood transfusion was collected from 7,337 medical records

**Figure 3.**
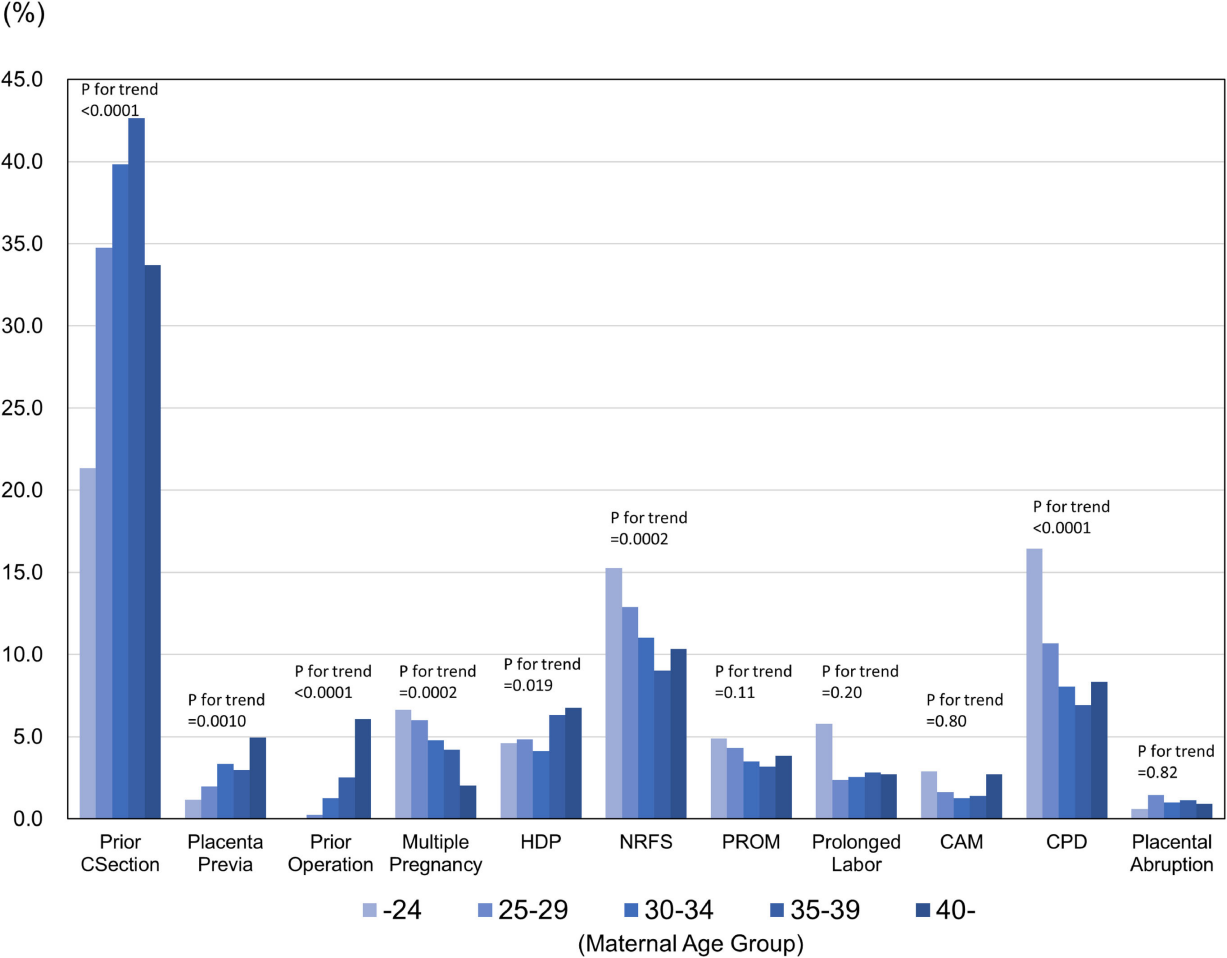
Indications for cesarean delivery according to age group (*n* = 5,506). CAM, chorioamnionitis; CPD, cephalopelvic disproportion; HDP, hypertensive disorders of pregnancy; NRFS, non-reassuring fetal status; PROM, premature rupture of membranes.

Delivery information according to age group is shown in Figure 2. The incidence of each mode of delivery was: spontaneous vaginal delivery, 61.5%; assisted delivery, 11.5%; and cesarean delivery, 23.5%. Older age groups exhibited a lower rate of vaginal delivery and a higher rate of cesarean section and blood transfusion (*P* < 0.0001). The indications for cesarean delivery were: prior cesarean delivery, 37.8%; NRFS, 11.1%; cephalopelvic disproportion, 8.8%; HDP, 5.1%; multiple gestation, 4.8%; PROM, 3.7%; placenta previa, 2.9%; prolonged labor, 2.8%; prior uterine surgery, 1.7%; chorioamnionitis, 1.6%; and placental abruption, 1.1% (Figure 3). Prior Cesarean section and operation were observed more often in the older age groups, whereas cephalopelvic disproportion were observed more often in younger age group (*P* < 0.0001).

Table 3 shows the birth information for 22,561 singleton liveborn infants. The mean gestational age at delivery was 38.7 (standard deviation [SD], 1.8) weeks. The proportion of premature delivery was 6.5% and post-term delivery was 0.1%. The mean birth weight was 3,024.1 (SD, 435) g and the proportion of low birth weight was 8.5%. The proportion of Apgar scores <7 was 10.4% at 1 minute and 10.4% at 5 minutes. The mean UApH was 7.31 (SD, 0.15), the proportion of UApH <7.2 was 6.51%, and the proportion <7.0 was 0.08%. Admission to the NICU was required in 5.14% of singleton newborns. Older age groups exhibited a higher rate of premature labor and NICU admission (*P* < 0.0001).

**Table 3.** Birth information from 22,561 singleton liveborn infants

Variables		Total			Maternal age at enrollment, years															*P* for trend

					≤24			25–29			30–34			35–39			≥40			
		*n*	(%, mean)	(SD)	*n*	(%)		*n*	(%)		*n*	(%)		*n*	(%)		*n*	(%)		
Gestational age at delivery, weeks	<37	1,474	6.5		121	5.7		340	5.4		488	6.0		367	7.5		158	13.7		<0.0001
	37–41	21,060	93.3		2,010	94.1		5,895	94.5		7,660	93.9		4,496	92.4		999	86.3		<0.0001
	>42	27	0.1		4	0.2		6	0.1		12	0.1		5	0.1		.			0.36
	Mean, SD	22,309	38.7	1.8	2,107	39.0	1.6	6,181	38.9	1.7	8,097	38.7	1.7	4,842	38.5	1.9	1,082	38.3	2.1	<0.0001

Birth weight	Mean, SD	22,311	3,024.4	435.0	2,108	3,037.1	415.1	6,180	3,032.7	412.5	8,100	3,023.7	431.9	4,841	3,016.7	462.5	1,082	2,991.7	489.0	0.028
Length	Mean, SD	22,039	49.3	3.8	2,075	49.4	2.2	6,085	49.4	2.4	8,006	49.3	5.5	4,802	49.2	2.7	1,071	49.0	3.0	0.0002
Head circumference	Mean, SD	22,019	33.4	2.5	2,075	33.2	1.5	6,082	33.3	1.5	8,000	33.4	3.5	4,791	33.5	1.6	1,071	33.5	1.8	<0.0001
Chest circumference	Mean, SD	21,984	31.8	2.8	2,070	31.8	1.8	6,074	31.9	1.8	7,989	31.9	3.8	4,781	31.9	2.0	1,070	31.8	2.4	0.78
Sex	Male	11,553	51.8		1,123	53.2		3,242	52.4		4,197	51.8		2,465	50.9		526	48.6		0.0052
	Female	10,764	48.2		985	46.7		2,942	47.6		3,905	48.2		2,376	49.1		556	51.4		0.0050
	Unknown	6	0.0		1	0.0		2	0.0		1	0.0		2	0.0		.			0.66

Placental weight	Mean, SD	20,058	562.6	156.1	1,935	572.1	242.7	5,451	560.8	124.9	7,213	561.5	159.0	4,456	563.2	139.6	1,003	558.6	142.9	0.33

Apgar score <7 at 1 min	+	2,348	10.4		165	7.7		710	11.4		894	11.0		425	8.7		154	13.3		0.40
	−	20,213	89.6		1,970	92.3		5,531	88.6		7,266	89.0		4,443	91.3		1,003	86.7		
Apgar score <7 at 5 min	+	2,343	10.4		181	8.5		748	12.0		905	11.1		367	7.5		142	12.3		0.016
	−	20,218	89.6		1,954	91.5		5,493	88.0		7,255	88.9		4,501	92.5		1,015	87.7		

UmA pH	Mean, SD	17,226	7.31	0.15	1,696	7.30	0.17	4,689	7.31	0.12	6,128	7.31	0.15	3,811	7.31	0.08	902	7.29	0.34	0.47
	<7.00	45	0.26		6	0.36		12	0.26		20	0.33		3	0.08		4	0.44		0.35
	7.00 to ​ <7.20	1,095	6.36		118	6.96		313	6.68		360	5.87		245	6.43		59	6.54		0.38
	≥7.20	16,086	93.38		1,572	92.69		4,364	93.07		5,748	93.80		3,563	93.49		839	93.02		0.29

NICU admission	+	1,159	5.1		96	4.5		265	4.2		409	5.0		300	6.2		89	7.7		<0.0001
	−	21,402	94.9		2,039	95.5		5,976	95.8		7,751	95.0		4,568	93.8		1,068	92.3		

NICU, Neonatal intensive care unit; SD, standard deviation; UmA, umbilical artery.

Table 4 shows the birth outcomes of 542 twin liveborn infants. A dichorionic diamniotic (DD) pregnancy was noted in 63.1%, monochorionic diamniotic (MD) in 35.8%, and monochorionic monoamniotic (MM) in 1.1%. The mean gestational age at delivery was 35.2 (SD, 2.6) weeks. Premature delivery was noted in 68.3%. The mean birthweight was 2,190.1 (SD, 474.7) g, and the proportion of low birth weight was 76.0%. The proportion of Apgar scores <7 was 12.9% at 1 minute and 9.2% at 5 minutes. The proportion of infants with UApH <7.2 was 5.0%, and 56.1% of twin newborns were admitted to the NICU.

**Table 4.** Birth outcomes in 542 twin liveborn infants

Variables			Total			Maternal age at enrollment, years															*P* for trend

						≤24			25–29			30–34			35–39			≥40			
		*n*	*n*	(%, mean)	(SD)	*n*	(%)		*n*	(%)		*n*	(%)		*n*	(%)		*n*	(%)		
Chorionicity	DD	542	342	63.1		26	56.5		82	56.2		124	63.3		98	73.1		12	60.0		0.011
	MD		194	35.8		18	39.1		64	43.8		70	35.7		34	25.4		8	40.0		0.015
	MM		6	1.1		2	4.4		0	0.0		2	1.0		2	1.5		0	0.0		0.59

Gestational age at delivery, weeks	<37	542	370	68.3		36	78.3		98	67.1		136	69.4		88	65.7		12	60.0		0.18
	37–41		172	31.7		10	21.7		48	32.9		60	30.6		46	34.3		8	40.0		0.18
	Mean, SD		542	35.2	2.6	46	34.2	3.5	146	35.5	2.0	196	35.0	2.8	134	35.3	2.5	20	35.8	1.6	0.0032

Birth weight	Mean, SD		542	2,190.9	474.7	46	2,057.4	571.3	146	2,199.4	398.5	196	2,180.9	501.5	134	2,224.7	479.9	20	2,305.8	413.3	0.032
Length	Mean, SD		524	45.0	3.9	44	44.1	4.8	138	44.9	3.3	192	44.9	4.1	130	45.4	3.6	20	46.5	3.3	0.0041
Head circumference	Mean, SD		520	31.7	2.2	44	31.0	3.1	144	31.7	2.0	195	31.5	2.6	130	32.0	1.8	20	32.1	1.8	0.018
Chest circumference	Mean, SD		518	28.5	2.7	44	27.4	3.2	136	28.5	2.3	190	28.4	3.0	128	28.9	2.3	20	28.7	2.0	0.048
Sex	Male	542	272	50.2		21	45.7		74	50.7		99	50.5		71	53.0		7	35.0		0.92
	Female		270	49.8		25	54.3		72	49.3		97	49.5		63	47.0		13	65.0		0.92

Placental weight	Mean, SD		484	913.2	320.9	40	988.5	265.9	122	923.5	325.6	178	867.6	291.9	124	930.0	353.0	20	1,002.4	393.8	0.80

Apgar score <7 at 1 min	+	542	70	12.9		8	17.4		19	13.0		24			19	14.2		.			0.32
	−		472	87.1		38	82.6		127	87.0		172	87.8		115	85.8		20	100.0		
Apgar score <7 at 5 min	+	542	50	9.2		5	10.9		16	11.0		15	7.7		14	10.4		.			0.36
	−		492	90.8		41	89.1		130	89.0		181	92.3		120	89.6		20	100.0		

UmA pH	Mean, SD		449	7.34	0.05	39	7.34	0.04	119	7.34	0.04	165	7.34	0.05	106	7.33	0.04	20	7.32	0.05	0.017
	<7.00	458																			
	7.00 to ​ <7.20		4	0.87		0	0.00		1	0.81		2	1.19		0	0.00		1	5.00		0.47
	≥7.20		454	99.13		39	100.00		123	99.19		166	98.81		107	100.00		19	95.00		0.47

NICU admission	+	542	304	56.1		32	69.6		90	61.6		104	53.1		67	50.0		11	55.0		0.011
	−		238	43.9		14	30.4		56	38.4		92	46.9		67	50.0		9	45.0		

DD, dichorionic diamniotic; MD, monochorionic diamniotic; MM, monochorionic monoamniotic; NICU, neonatal intensive care unit; SD, standard deviation; UmA, umbilical artery.

Gestational age at the time of maternal blood collection (*n* = 22,356) is shown in supplementary data (eFigure 1) and gestational age at the time of cord blood collection (*n* = 18,874) is demonstrated in eFigure 2.

## DISCUSSION

The present study describes the maternal baseline characteristics and perinatal outcomes in participants of the TMM BirThree Cohort Study.^19^ The TMM BirThree Cohort Study is expected to provide scientific evidence to assess risk factors for various multifactorial diseases, including NCDs, by analyzing genetic-environmental interactions. Specifically, this cohort study is accumulating genetic information from SNP arrays or whole-genome sequencing,^29^ epigenetic information from targeted bisulfite sequencing, and quantitative metabolome data by nuclear magnetic resonance- or mass spectrometry.^30^ Precise clinical information is being collected from medical records, and epidemiological data is being obtained by questionnaires and official government databases. Accordingly, this cohort study will enable a variety of genome-metabolome-wide association studies of obstetric and pediatric complications. In addition, this cohort study will reveal associations of exposures in early life with the onset of NCDs in later life, including developmental, cognitive, metabolic, and vascular disease. Finally, this cohort study will establish unexplored methods of early prevention using personalized genetic, epigenetic, medical, and epidemiologic information obtained from multiple generations. Recently, birth cohort studies have been encouraged to move forward the concept of data sharing, aiming to enable cross-comparisons or meta-analyses of integrated data at the regional, national, and global level. Our study aims to provide precise perinatal information to align with those agendas.

We found that approximately 6.6% of patients used assisted reproductive technology, comparable with nationwide Japanese data.^31^ These data will allow us to conduct association studies of the effect of assisted reproductive technology on developmental outcomes, from not only an epidemiologic viewpoint, but also from a molecular biological viewpoint.

Maternal infection screening tests stratified by maternal age demonstrated that an overview of series of valuable results for the birth cohort study. Further transgenerational epidemiological analysis in our cohort study might provide an informative information to develop methods for the earlier intervention. Detailed information on the rate of maternal infections that have a potential for fetal transmission will help to establish reference data for the Japanese population of women of reproductive age.

Landscape obstetric outcome data stratified by maternal age group were demonstrated in the present study. The diagnostic criteria for all major obstetric complications are standardized at the participating facilities of Tohoku University and by clinical guidelines in Japan.^32^ We suggest the construction of a phenotyping algorithm for subtypes of HDP, using the huge trove of outpatient data available from the BirThree cohort on blood pressure, urinalysis, and other maternal and fetal factors. Standardized phenotyping would empower the implementation of high-resolution association studies. Advanced maternal age clearly exhibited a significantly higher rate of low lying placenta, PP, GDM and HDP. These results were in agreement with previous reports,^21^ and biological mechanisms would be revealed by multi-omics analysis using samples from three generations in our cohort study.^19^

The major strength of our study is that it provides precise clinical information obtained from medical records, and that the diagnoses employ standardized criteria. Most of participants had a “Maternal and Child Health Handbook” of their own, in which they recorded their mothers pregnancy details and birth outcomes. Additionally, family information from partners, grandparents, siblings, and others was available for transgenerational analyses.^19^ Another strength lies in the systemic collection of biospecimens, including blood and urine samples in early and midpregnancy, umbilical cord blood, and breast milk. Blood samples were stored in the biobank as plasma, serum, and immortalized lymphocytes for future research.^26^ Genome analysis by Japonica array was performed in most maternal samples,^29^^,^^33^ allowing us to analyze GWAS for various parameters, variables, and diseases. We are eager to share our data to enable meta-analyses and international collaboration.

Several limitations should be considered in this study. Participants were recruited between 2013 and 2017 in Miyagi Prefecture, located in northern Japan. Therefore, the ability of the cohort to represent the national population could be discussed. Second, the long-term effect of the GEJE disaster in 2011 may affect baseline characteristics and epidemiologic outcomes. Extensive epidemiologic surveys and omics analyses would elucidate the mechanisms of various factors of the disaster.

In conclusion, the present study clearly and precisely demonstrates the perinatal baseline profiles in participants of the TMM BirThree Cohort Study.^19^ The data presented in the study are expected to provide strategic information for various types of association studies to uncover perinatal or developmental diseases, including NCDs, in later life through genomic-environmental interactions. Collaborative data visiting or sharing will bring new insight into the search for personalized early prevention and intervention strategies.

## APPENDIX A. SUPPLEMENTARY DATA

eFigure 1. Gestational age at the time of maternal blood collection (first time point, *n* = 22,449; second time point, *n* = 22,356)

eFigure 2. Gestational age at the time of cord blood collection (*n* = 18,874)
