# Supplementary material for: Maternal Baseline Characteristics and Perinatal Outcomes: The Tohoku Medical Megabank Project Birth and Three-Generation Cohort Study
Source: J Epidemiol. 2022 Feb 5;32(2):69–79. doi: 10.2188/jea.JE20200338 (PMC8761563; doi:10.2188/jea.JE20200338)
Supplement: Supplementary file 1 [file je-32-069-s001.pdf]

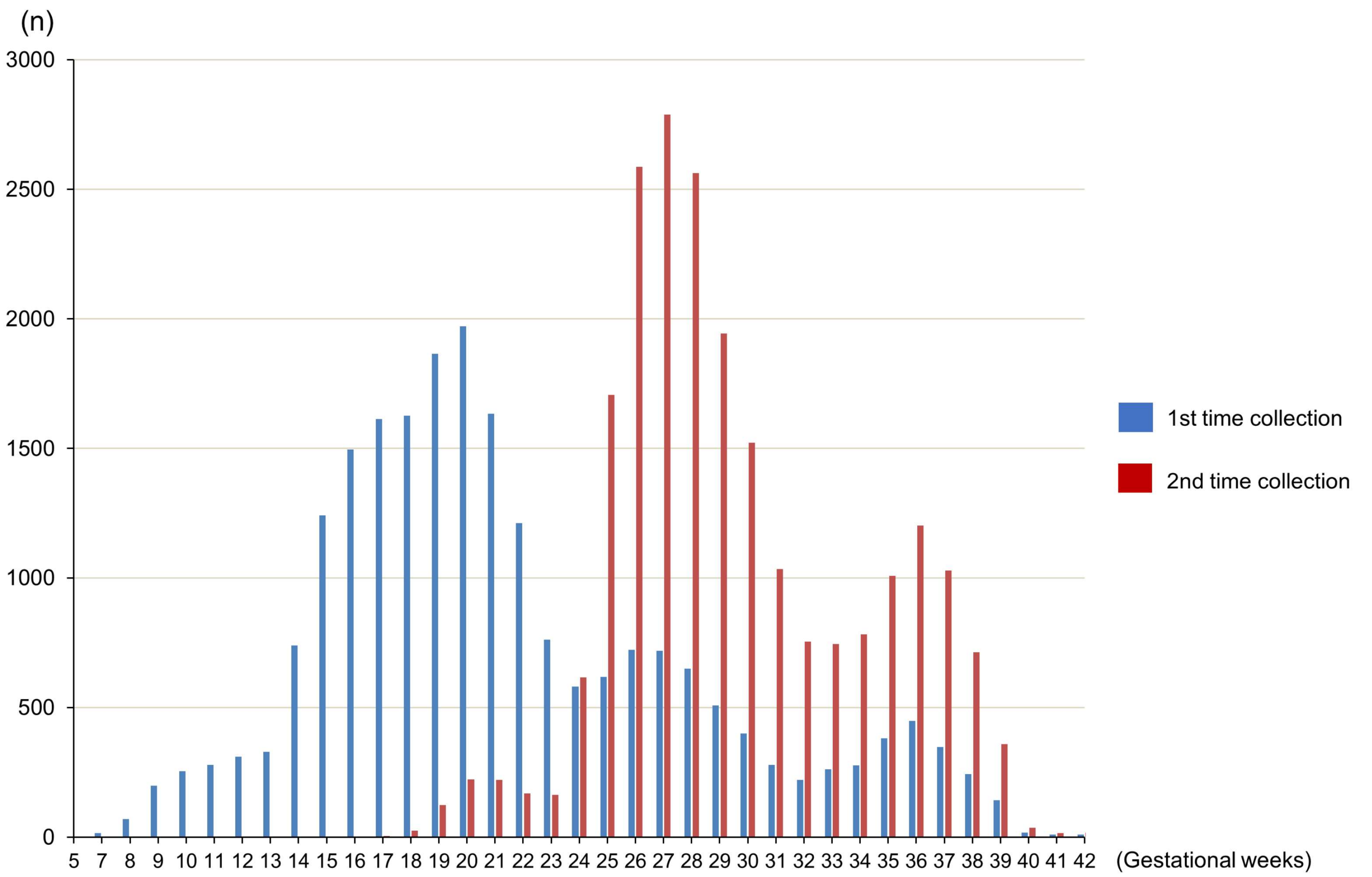

eFigure 1.
Gestational age at the time of maternal blood collection
(first time point, n=22,449; second time point, n=22,356)

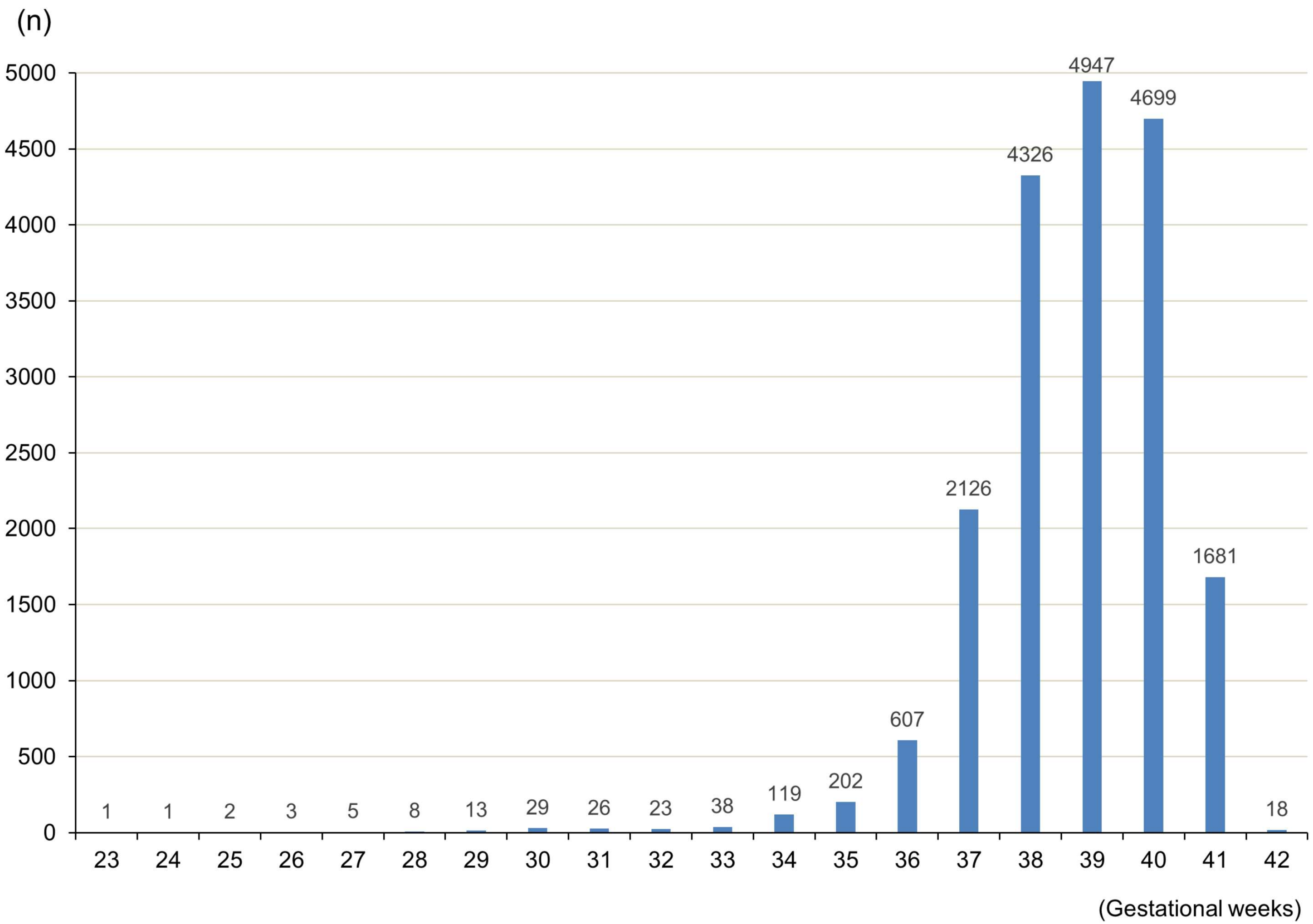

eFigure 2.
Gestational age at the time of cord blood collection (n=18,874)
